# Food-Borne Disease Prevention and Risk Assessment

**DOI:** 10.3390/ijerph17145129

**Published:** 2020-07-16

**Authors:** Ewen Todd

**Affiliations:** Ewen Todd Consulting LLC, Okemos, MI 48864, USA; todde@msu.edu

“Food-borne Disease Prevention and Risk Assessment” is a Special Issue of the *International Journal of Environmental Research and Public Health* on understanding how food-borne disease is still a global threat to health today and to be able to target strategies to reduce its prevalence. Despite decades of government and industry interventions, food-borne disease remains unexpectedly high in both developed and developing nations. For instance, the Centers for Disease Control and Prevention (CDC) estimates that one in six persons in the United States suffers from gastroenteritis each year, with up to 3000 fatalities arising from consumption of contaminated food [1]. According to the WHO Initiative to Estimate the Global Burden of Food-borne Diseases, 31 global hazards caused 600 million food-borne illnesses and 420,000 deaths in 2010; diarrheal disease agents were the leading cause of these in most regions caused by *Salmonella,* but *Taenia solium*, hepatitis A virus, and aflatoxin were also significant causes of food-borne illness [2,3]. The global burden of food-borne disease by these 31 hazards was 33 (95% UI 25–46) million Disability Adjusted Life Years (DALYs) in 2010; 40% of the food-borne disease burden was among children under five years of age. Since we know that most food-borne diseases are preventable, these are astonishing figures for the 21st century. We are familiar with some of the underlying conditions: unsafe water used for the cleaning and processing of food, poor food-production processes, inadequate storage, and food-handling practices including infected food workers and cross-contamination of food. These can be coupled with inadequate or poorly enforced regulatory standards and industry compliance. However, knowledge of these is not enough. Making advances in prevention and control practices requires a suite of interlinked actions from improvements in the investigation of complaints and illnesses to finding the root cause of outbreaks; applying rapid and accurate identification of the hazards present; determining the conditions in which pathogens grow and multiply in order to eliminate or reduce these numbers; developing targeted intervention strategies; understanding human behavior with respect to food processing and its preparation; producing effective educational and training programs; evaluating the risks of existing and modified food production and preparation practices; predicting how effective potential interventions would be, and introducing effective and enforceable codes of practice for the different harvesting, processing, and preparing industry components. The human element is now known to be critical in applying safe practices to prevent food-borne illnesses, but it is much more difficult to influence for positive change, both from the culture of an organization and individual backgrounds and preferences. This issue is a modest attempt to explore some of these efforts through five publications.

Most agents causing food-borne illness have been identified over the last 145 years, starting from the pioneering work of Robert Koch who identified the cause of anthrax, tuberculosis and cholera. He also dismissed the then-current concept of spontaneous generation, used agar as a base for growing bacteria, and proposed his four postulates: (1) the organism must always be present, in every case of the disease; (2) the organism must be isolated from a host containing the disease and grown in pure culture; (3) samples of the organism taken from pure culture must cause the same disease when inoculated into a healthy, susceptible animal in the laboratory; (4) the organism must be isolated from the inoculated animal and must be identified as the same original organism first isolated from the originally diseased host. Over time, however, the rigid application of these postulates probably hindered research into the discovery of new agents, particularly viruses which initially could not be seen or isolated in culture. Today, nucleic acid-based microbial detection methods have made Koch’s original postulates less relevant, because these methods make it possible to identify microbes associated with a disease, even if they are non-culturable. Prions are another class of agents that do not fit into the classical infectious disease agent being misfolded proteins with the ability to transmit their misfolded shape onto normal variants of the same protein to cause transmissible neurodegenerative diseases in humans and some animals. Thus, a challenge today is to be prepared to identify and characterize new infectious agents which can arise from unexpected sources. This applies to coronaviruses which have recently been brought to the public’s attention where humans have been infected from animal sources. These include severe acute respiratory syndrome coronavirus (SARS-CoV), for which bats are a major reservoir of many strains, and other strains have been identified in palm civets; Middle East respiratory syndrome-related coronavirus (MERS-CoV), is a species of coronavirus which also has reservoirs in bats, and but has spread to camels and from there to humans, particularly camel handlers; and the current COVID-19 virus pandemic affecting millions of people worldwide, which likely originated from wet markets in Wuhan, China, where domestic and wild animals are slaughtered for customers; however, significantly, bats may also be the primary reservoir. 

This background makes the paper of Wen, Sun, Li, He and Tsai [4], *Avian Influenza—Factors Affecting Consumers’ Purchase Intentions toward Poultry Products*, all the more relevant for those seeing increasing links between animals and human diseases. Influenza viruses, belong to the Orthomyxoviridae, a different family from the coronaviruses; yet, strains of both of these infect humans and animals, and some can be transmitted from animals to humans; these include the H1N1 avian influenza (swine flu) of 2009, which killed between 151,000 and 575,000 people worldwide) and H5N1, strain (popularly known as the bird flu) which had pandemic potential. In particular, poultry production and sales have led to the spread of H5N1 and other avian influenza viruses [5]. This strain was first isolated from a goose in China in 1996 and it spread throughout Asia and Europe over the next decade with associations of wild birds and poultry. Large sums of money were spent in order to eliminate this disease despite the relatively few associated human illnesses and deaths worldwide, and most Europeans who had limited exposure to H5N1 feared any new viruses such as the avian flu and avoided uncooked chicken products [6]. Poultry production dropped 25-30% in many Asian countries, including China. A subsequent avian influenza strain, A H7N9, also caused human infections although the number of human cases transmitted by this strain was more limited than for H5N1. Nevertheless, populations in Asia and particularly China have been sensitized to the potential risks of human infections and economic damage from news’ reports of avian influenza. The paper of Wen et al. [4] focuses on the purchase intentions consumers in Guangzhou, China, during recurring reports of this epidemic. Avian influenza A H7N9 virus had not previously been seen in either animals or people until it was found in March 2013 in China. However, since then, infections in both humans and birds have been observed, and the disease is of concern because most patients have become severely ill. Most of the cases of human infection with this avian H7N9 virus were associated with recent exposure to live poultry or potentially contaminated environments, especially markets where live birds have been sold. This virus does not appear to transmit easily from person to person, and sustained human-to-human transmission has not been reported. However, according to the Food and Agriculture Organization (FAO) [7], case-control studies suggest contact with poultry or a visit to a live poultry market in the two weeks prior to disease onset was a significant risk factor. Cases have been reported in humans who visited live bird markets, slaughtered poultry or pigeons, transported poultry, and brought live poultry into their homes. As of December 2019, the number of confirmed human cases and deaths was 1568 and 616, respectively, and 26 live markets in 15 Chinese provinces tested positive for the virus, mainly in chicken samples [8]. Thus, it is understandable that Chinese purchasers of recently slaughtered poultry should have concerns for their health, and they would consider avoiding purchasing any chicken products. 

Wen et al. [4] found, unsurprisingly, that from a risk perception perspective, the more consumers believed purchasing chicken products was a risk during a period of this avian influenza outbreak, the more they reduced their purchase of chicken products, since they had low levels of trust in the quality of chicken meat. Since the public receives most of its information on avian influenza and its relationship to human illness, animal diseases and food contamination, through the mass media as it is narrated and shown to consumers, will influence and change their willingness to purchase chicken products. The authors recommended that government provides accurate information on the public health system to ensure the stable and healthy development of the poultry meat products or consumers, and to rebut any misleading media reports. However, this depends on how much trust the people are willing to place on government agencies. As Bánáti [6] indicates, there was distrust in the past in industry and government oversight of the food supply developed because of food scares such mad cow disease, dioxin in pork, melamine in pet and baby food, and now more recently in outbreaks of avian influenza, and the current COVID-19 pandemic. Although coronaviruses, particularly COVID-19, are not food-borne, the worldwide public may be overly cautious about any food they purchase and wet markets in Asia may see a drop in attendance at least until such pandemics are over. It would be interesting to explore how long anxiety over food purchases occur after this pandemic is over, but it seems the longer they last through media coverage, the more the concern will remain.

The second paper in this series, entitled *Cognitive Biases of Consumers’ Risk Perception of Food-borne Diseases in China: Examining Anchoring Effect* by Shan, Wang, Wu and Tsai [9], also focuses on the perception of risks of food-borne illness in China. The authors indicate that the home is the place where the largest number of food-borne illness cases occur in China, and one of the reasons for this is that many consumers are not aware of their vulnerability to such illnesses and they underestimate their risk. This seems to be opposite to the findings of Wen et al. [4] where consumers are very concerned about avian influenza transmission, but the contrast can be explained because there is virtually no media coverage of food-borne illnesses at home. Because consumers seem to have limited knowledge of the risks, the authors propose that they tend to use an anchoring strategy on which to base their food-borne disease prevention and control decisions. The authors argue that since consumers are not always rational in making decisions, they often adjust their judgments on their subjective understanding and their initial reference information (called the initial anchor). However, other factors such as an uncertain external environment and limited knowledge make consumers unsure of the extent to which they can adjust their estimates. These limitations in information processing result in biased anchoring results, which they call the “anchoring effect”. The authors postulate that because Chinese citizens have limited scientific literacy compared with those in developed countries, Chinese consumers should have significant cognitive biases including the anchoring effect. Although there are few reports on whether there is an anchoring effect in consumers’ risk perception of food-borne disease, previous studies of other diseases have confirmed that there is indeed an anchoring effect, such as overestimating the risks of breast cancer. To test whether or not consumers’ limited knowledge results in a significant anchoring effect, the authors collected survey data from 375 consumers in Wuxi, Jiangsu Province. A questionnaire obtained information on how much the respondents knew about food-borne diseases and how they could be prevented. Based on the approximate national food-borne disease prevalence rate of 15% of the population, in this study 30% and 5% food-borne disease prevalence were selected as high and low anchor values, respectively. This experimenter-provided anchor value, a history of food-borne disease, and familiarity with those diseases were found to be important factors influencing the respondents’ anchoring effect. They found that when more information was provided to the respondents in the study (considered as a short-term intervention), their risk perception was improved to some extent, but there were still anchoring biases. As a result, Shan et al. [9] argue that short-term interventions would not substantially change consumers’ anchoring effect, and there is a need for stronger and more long-term interventions. They recommend that government should play an active role in publicity and education aimed at the public about food-borne diseases. Specifically, the prevalence and scientific context about different food-borne diseases should be disseminated to consumers through various media, such as the internet, television, and radio, to warn consumers of the objective risks of these diseases. Therefore, they argue that improving consumers’ risk perception of food-borne disease is critical to the long-term prevention of illness from these risks. They concluded that government should strengthen active monitoring, publicity, and education about food-borne disease, so that individuals are more knowledgeable scientifically to improve their perception in making judgments about risks of food-borne disease. However, knowledge alone may not be enough. Da Cunha et al. [10] found that education is not as effective as training in school food handlers in Brazil. Rossi et al. [11] observed that although food handlers have knowledge of microbiological risks, their risk perception has a weak association with food safety knowledge. They stated that, unfortunately, food handlers demonstrate an awareness of food safety, but they generally fail to translate that knowledge into safe practices because of their optimistic bias. Optimistic bias is a psychological phenomenon in which people believe they are less likely to experience adverse events than others, such as in home-prepared meals. This concern also applies to consumers eating out; they can incorporate a sense of affection and identity to a place, associating it with making their own meals at home, and do not identify the risk of food-borne disease while eating at those restaurants [12]. Like food handlers, consumers have a feeling of overconfidence in the restaurant they eat with their optimistic bias. This result reinforces the need for governments and health agencies to protect the health of the population. Wildemann [13] also points out that although food-borne illnesses contribute substantially to the overall burden of disease, including hospitalizations, economic loss, and death, in contrast to food safety experts, the public usually perceives food-borne diseases as low risk. This distinguishes the differences in the perception of the risk between experts and the public. Wildemann [13] lists many qualitative factors affecting risk perception and evaluation. These include mild symptoms vs. potential fatal consequences or delayed adverse effects; dread or low concern for a certain disease; reversibility of the effects of the disease (e.g., long-term sequelae, reduced quality of life, or rapid recovery); previous history of the disease in the family or community; existing health of the individual, e.g., immunocompromised; familiarity of the agents or disease and understanding its means of transmission; increasing or decreasing public concern; exposure and impact controllable; risk determined by personal actions or mistakes made by others; trust in institutions; much or little media attention to the concern. Rosati and Saba [14] found that the concern about food risks was found to be statistically significantly dependent on the perception of risk to the individual. Usually, food-borne illness will not evoke outrage among lay people because they are perceived as voluntary, controllable, visible, and familiar. This means that most individuals perceive the threats of food-borne diseases as low, although food can pose significant risks. In particular, food-borne illness originating in the home is perceived as familiar and controllable. 

For Wuxi consumers and, by extrapolation, for Chinese residents on the whole, there should be a low perceived risk even though the prevalence of food-borne disease in China is as high as 15%. This is similar to the percentage in the USA (17%) where, according to Scallan et al. [1], one in six persons is estimated to suffer from food-borne illness each year. Wildemann [13] emphasizes among the factors associated with increased concern are high media attention, and any risk message and its originator are crucial components for informing the public what actions to take of any food-borne disease concern; she emphasizes that if the public does not consider the source credible, it will be difficult to convey the message and effect long-term changes in attitude. This seems to be consistent with a long-term-held anchoring effect described by Shan et al. [9]. Credibility has two dimensions: expertise and trustworthiness. Expertise refers to the knowledge in a specific area and trustworthiness to the reliability of the message content. Trust depends on three factors: knowledge expertise, honesty concerning the completeness of the provided information, and whether the concerns of the consumers are taken seriously or not by the risk message originator [14]. Therefore, trust plays a major role in the credibility and acceptance of an institution to influence the processing of risk information and potential changes in consumer behavior. Involving the media during the whole process may enhance the trust of the public in food safety policy. All this information questions whether it is possible, without extensive government media campaigns and perhaps a scare factor like avian influenza in a population, to substantially change attitudes and behaviors towards food safety through reducing the anchoring effect. Unfortunately, although food scares draw public attention, they can also create false or misleading information that has to be countered by the experts [6], and the public may become polarized between being ultra-protective of personal and family health to a cavalier attitude to throw caution to the wind, as seems to be the case in the current COVID-19 pandemic.

The discussion on perception and communication of risk and how translate government polices into changed behavior takes us to the third paper in this issue, that of Farias, Akutsu, Botelho, and Zandonadi [15] discussing *Good Practices in Home Kitchens: Construction and Validation of an Instrument for Household Food-Borne Disease Assessment and Prevention*. The purpose of the study was to develop and validate an instrument to evaluate Brazilian home kitchens’ good practices. the rationale for this was for food preparers at home to avoid food-borne diseases illnesses by adopting preventive actions throughout the home food production chain. Although governments regulate food safety practices in commercial food production and food service establishments, there are no regulations on how to control food preparation and handling in the home. From the work of Rossi et al. [11] and Shan et al. [9], consumers may have an optimistic bias that creates an anchoring effect to fix consumers’ the risks associated with food-borne illness. Therefore, there needs to be more information on how to reduce food-borne domestic cases through improving food handling practices. After the instrument was developed, the content was validated using the Delphi technique with independent food hygiene and food safety specialists, and a focus group for validation of the criteria. The study showed that consumers in Brazil tend not to perceive themselves, or someone in their family, to be susceptible to food-borne illness; rank their risk of food-borne illness lower than that of others; and/or do not follow all recommended food safety practices, and, consequently, they do not take sufficient precautions to prevent illnesses from occurring. The authors found that food was prepared in the home where there were heavily contaminated areas in the kitchen (refrigerator handles, tap handles, sink drain areas, dishcloths, and sponges) because it is unusual for these surfaces to be frequently washed or cleaned. Additionally, raw or unwashed foods were constantly touched during meal preparation. The authors state that because there is limited guidance for home food preparers, the use of an such an instrument helps evaluate the level of food safety at home, and identifies unsafe practices in food handling for targeted prevention and control strategies though improving consumer knowledge about food and waterborne diseases and their consequence. Farias et al. [15] certainly developed a method to comprehensively understand the risk of home food preparation in a Brazilian community and presumably would have global value for helping to reduce risks that have led to the annual estimate of 600 million food-borne illnesses worldwide [3]. Similar studies have been done in the past such as that of Redmond and Griffith [16] who said that knowledge, attitudes, intentions, and self-reported practices do not correspond to observed behaviors, suggesting that observational studies provide a more realistic indication of the food hygiene actions actually used in domestic food preparation. Only an improvement in consumer food-handling behavior is likely to reduce the risk and incidence of food-borne disease. So, the question remains that unless food preparers are motivated, it may be very hard to change perceptions of risk of illness to themselves or who they serve. As Collins [17] pointed out 23 years ago, only 50% of consumers were concerned about food safety, partly because of lifestyle changes affecting food behavior, with an increasing number of women in the workforce, limited commitment to food preparation, and a greater number of single heads of households. Then, as now, it may be that consumers appear to be more interested in convenience and saving time than in proper food handling and preparation. Fischer et al. [18] showed that while most consumers are knowledgeable about the importance of cross-contamination and heating in preventing the occurrence of food-borne illness, this knowledge is not necessarily translated into behavior. Potentially risky behaviors were observed in the domestic food preparation environment with errors like participants allowing raw meat juices to come in contact with the final meal. The authors stated that procedural food safety knowledge (i.e., knowledge proffered after general open questions) was a better predictor of efficacious bacterial reduction than declarative food safety knowledge (i.e., knowledge proffered after formal questioning). This suggests that motivation to prepare safe food was a better indicator of actual behavior than knowledge about food safety *per se*. Byrd-Bredbenner et al. [19] point out that adding food safety cues to food packages may be particularly effective given that nearly half of consumers indicate they commonly read cooking instructions on food packages. Moreover, some especially “teachable moments” are after publicized food-borne illness outbreaks or recalls, before major holidays, during the perinatal period, and after being diagnosed with an immune-compromising condition. However, providing food safety information for those at increased risk of poor food-borne illness outcome often is not part of standard clinical practice among health professionals, and role models like athletes do not always demonstrate good food safety practices.

The fourth paper takes the reader from understanding risk perception and risk communication strategies for prevention of food-borne illnesses in homes and restaurants to reviewing mathematical models to help risk managers in making decisions for reducing food-borne disease, in this case the beef industry. Risk assessments have been promoted to address specific issues with the impact of chemical contaminants in the health and environmental fields for over 70 years, but a standardized risk-based food safety management approach was only recommended and adopted by the Codex Alimentarius Commission of the World Health Organization (WHO) in the last 21 years [20]. This Commission defined risk analysis as comprising risk assessment, risk management, and risk communication, and all types of contaminants were considered, including microbiological ones which have specific modeling challenges in that pathogens can increase and decrease over the production, transport, storage, and preparation of foods. Microbiological risk assessment is a scientific evaluation that aims to provide an estimation of a risk considering the probability and the severity of health effects caused by a bacterial, viral or parasitic hazard in order to support decision-making processes. The Joint FAO/WHO Expert Meetings on Microbiological Risk Assessment (JEMRA) began in 2000 in response to requests from the Codex Alimentarius Commission and FAO and WHO Member Countries and the increasing need for risk-based scientific advice on microbiological food safety issues. Quantitative microbiological risk assessments (QMRAs) aim at determining the existing public health risk associated with biological hazards in a food using mathematical equations to estimate the change of microbial load after each processing step and then to compare the efficiency of different risk reduction measures [21]. Model inputs are generated by collecting data or soliciting experts. QMRA models comprise four steps: hazard identification, exposure assessment, hazard characterization, and risk characterization. QRMAs enable experts to estimate the risk to which the population may be exposed, evaluate possible risk mitigation strategies, and generate knowledge for the better management of risks associated with contamination events. The assessment involves measuring known microbial pathogens or indicators and running a Monte Carlo simulation throughout different steps in the food chain to estimate the risk of transfer from the food to the consumer. If a dose–response model is available for the microbe, it would be used to estimate the probability of infection.

The present study of Tesson, Federighi, Cummins, Mota, Guillou, and Boué [22], entitled *A Systematic Review of Beef Meat Quantitative Microbial Risk Assessment Models*, was to conduct a critical analysis of beef QMRAs to help identify present and future contamination challenges in beef production. The authors’ review was comprehensive with 67 publications selected, but the focus was limited to studies in western countries and for a limited number of pathogens, mainly Enterohemorrhagic *Escherichia coli* (EHEC) and *Salmonella* spp. The authors concluded from the QMRAs that there were sufficient public health risks associated with beef meat consumption that specific risk mitigation strategies must be put in place. Because it was difficult to compare the different models used in each study, it was not possible to rank risk mitigation strategies by study in terms of effectiveness or hazards in terms of priority. Nevertheless, the authors highlighted the major risk mitigation strategies. For instance, those for EHEC and *Salmonella* should have a priority on the reduction of their prevalence before slaughter, e.g., the shedding condition of the animal, and the reduction of cross-contamination on the product, e.g., pathogen dispersion during dehiding, and to a lesser effect during evisceration and splitting; this would be followed by rapid chilling of the carcass to prevent growth of these pathogens and to lessen contamination of the final beef products during fabrication. Because there are limited data on the potential for cross-contamination during transportation from the farm to the slaughterhouse or during holding in the lairage, this step in beef production is difficult to model without a high degree of uncertainty. However, because it is known that lessening the length of the transit and lairage time has been observed to reduce the stress in cattle, in combination of good cleaning procedures for transport trucks and at the lairage, shedding and cross-contamination of enteric pathogens can be reduced by these actions. As a result, it is not necessary to model the whole farm-to-fork chain when trying to address specific risk management questions. In contrast, the authors argue that the strategies to control *Listeria monocytogenes* should focus on storage steps at retail and at home with information to the consumer, instead of emphasizing all the efforts on the slaughterhouse. Figure 5 in the publication is a useful summary of the most critical points raised in each of the 67 studies with a breakdown by Farm (prevalence of pathogens in cattle feces and hide coats; shedding time), Processing (dehiding and chilling), Retail and Consumer (storage temperature); Consumer (cooking preference and host susceptibility).

The authors conclude that QMRA is a very powerful tool providing valuable insights to assist managers make decisions to reduce the risk of infections arising from consumption of pathogens in beef, but they agree that models can only provide estimations with a level of accuracy that depends on quality and consistency of data for input into these models. Where there are data gaps in the meat production chain from farm to fork, surveys and targeted research should be encouraged to generate the missing information, but data extraction from some of the farm-to-fork steps may be difficult or even almost impossible. Therefore, proposed risk mitigation interventions for these steps may be unrealistic and hence the hazard can remain. However, if the need is great, persistence can achieve positive results. For instance, data gaps were explored to understand why deli meats sliced and packaged in the deli were contaminated with *Listeria monocytogenes* five to seven times more frequently than deli meats sliced and packaged by a processor [23]. Extensive testing and observations of worker behavior showed that these deli meats tended not to contain added inhibitors; resident *L. monocytogenes* were present in niches in equipment and spread through cross-contamination from food contact and non-food contact surfaces; and there was lack of adequate sanitation; inadequate temperature control; and inappropriate glove/hand issues. This information was used to create a “virtual deli” model and to generate six baseline situations and 22 scenarios by the U.S. Department of Health and Human Services; Center for Food Safety and Applied Nutrition, Food and Drug Administration and the U.S. Department of Agriculture Food Safety and Inspection Service [24,25,26]. Overall, the virtual deli model indicated that the greatest risk was from contamination present in an incoming chub of a product that permitted growth of *Listeria*. Even products that did not permit growth could still be a significant contributor to listeriosis, from environmental contamination and subsequent cross-contamination to other products. Important environmental factors contributing to risks were worker behaviors, the slicer construction and its maintenance, trash handling, and cleanup operations. The level of contamination at retail delis was found to directly affect the risk, where a two-fold decrease in contamination would result in a 20% reduction in illnesses. The simulation showed that if all deli meat products would have growth inhibitors coupled with appropriate control of temperature and storage time at the consumer’s home there would be fewer cases of listeriosis attributable to deli meats.

In another study, to acquire useful data for the consumer phase of a typical QMRA in the Netherlands, Chardon and Swart [27] designed a food consumption and food handling survey that was specifically aimed at obtaining quantitative data at the consumer level, typically not otherwise available. For a broad spectrum of food products, the survey covered the following topics: processing status at retail, consumer storage, preparation, and consumption. The final result was a coherent quantitative consumer phase food survey and parameter estimates for food handling and consumption practices in the Netherlands, including variation over individuals and uncertainty estimates. For instance, the survey showed that an average 40% of the fresh meat was stored in the refrigerator, 44% was stored in the freezer, and 18% of the dried sausages and 30% of the eggs were stored at room temperature. The mean storage time in the refrigerator was between 2 and 3 days for fresh meat and fresh meat products and about 4 days for cooked meat products and pâté. For understanding the risks of cross-contamination, 66% of chicken breasts were cut at home, and home-cut ingredients were added to 72% of precut lettuce. When meat and lettuce were prepared at the same time, 52% of the meat was cut before cutting the lettuce. Fortunately, rare and raw preparations of meat products were preferred by only 1 to 5% of the respondents; medium and done cooked food was the preference of the vast majority of those surveyed. However, 8% of respondents consumed steak tartare raw. However, more detailed information is needed on consumer preferences. For instance, products can be fresh or deep-frozen, meat cuts can be intact or consist of combined meat pieces, and beef can be mechanically tenderized with needles; not all these differences are known or acknowledged by consumers for food safety concerns. 

It is not always necessary to conduct a full QRMA to achieve a risk management goal for meat production. Pointon et al. [28] used qualitative risk assessments and expert opinion to develop a framework for profiling and managing risks associated with red meat-borne food safety hazards. Inputs included known ruminant food-borne pathogens *Clostridium perfringens*, *Campylobacter jejuni*, enterohemorrhagic *Escherichia coli* and *Salmonella* spp.; increase the shedding and transmission of pathogens by co-mingling of animals, as well as intensive rearing methods and stress (such as starvation and transport). The risk profile showed that there were low-risk ratings for pathogens in raw meats (products with a terminal cooking step) and for cooked cured meats. Uncooked comminuted fermented meats (UCFM) were ranked as low risk when the process was adequate enough to inactivate the expected loading of pathogens on incoming raw ingredients. Risk ratings were higher for *L. monocytogenes* in ready-to-eat meat products, for *Salmonella* in kebabs and for enterohemorrhagic *E. coli* and *Salmonella* in UCFM where the process was not adequate to inactivate these hazards in raw materials.

QMRAs can be combined with the use of the Codex Alimentarius’ newly adopted risk management metrics to improve public health outcomes. By estimating the food safety objective (the maximum frequency and/or concentration of a hazard in a food at the time of consumption) and the performance objective (the maximum frequency and/or concentration of a hazard in a food at a specified step in the food chain before the time of consumption), risk managers will have a better understanding of the appropriate level of protection (ALOP) from microbial hazards for public health protection. Crouch et al. [29] explored such a combination that allows identification of an ALOP and evaluation of corresponding metrics at appropriate points in the meat food chain with the example of a Monte Carlo QMRA for *Clostridium perfringens* in ready-to-eat and partially cooked meat and poultry products. For demonstration purposes, the QMRA model was applied specifically to hot dogs produced and consumed in the United States. Evaluation of the cumulative uncertainty distribution for illness rate allows a specification of an ALOP that, with defined confidence, corresponds to current industry practices; ALOPs considered were 13–21 *C. perfringens* illnesses per million servings of hot dogs where the prevalence of the pathogen in hot dog servings would be 0.72–1.76%.

The last of the five papers in this Issue, *Prevention and Control of Food-borne Diseases in Middle-East North African Countries: Review of National Control Systems* is by Faour-Klingbeil and Todd [30] who discuss how a region, in this case Middle-East North African (MENA) countries, tackles prevention and control of food-borne diseases, where for the most part there are limited industry and governmental scientific and economic resources. Most of this region is arid with limited rainfall that impacts agriculture and much of the food has to be imported. The 14 WHO global subregions have considerably different burdens of food-borne disease, with the greatest falling on the subregions in Africa, followed by the subregions in South-East Asia and the Eastern Mediterranean subregion because of adverse environmental and economic conditions. More specifically, one reason why some parts of the world suffer more from food and waterborne diseases is that the public health structure may be compromised, and their prevention and control strategies, including their regulatory standards, local enforcement, educational programs, surveillance and epidemiological information systems, and applied research towards advanced technologies, are less well developed [31]. The WHO Eastern Mediterranean Region contains most of the MENA countries with an estimated 100 million people living in this region suffering from food-borne illness, mainly from nontyphoidal *Salmonella, E. coli*, norovirus, and *Campylobacter* [3]. Despite most of these countries having similar cultures, there are great economic disparities among them with Yemen and Palestinian Gaza existing in extreme poverty at one end compared Gulf countries flush with oil revenues at the other. Several MENA countries have had histories of civil wars, some on-going as in Libya, Syria and Yemen. Over the years, many of these countries have the interest, but not the will to modernize their food safety oversight systems. The authors suggest that they should manage their national food safety programs based on risk analysis with an integrated farm-to-table approach [32], and use the Codex Alimentarius Commission (CAC) working principles, and the Procedural Manual [19], and Guidelines for National Food Control Systems (NFCS) comprising of Laws and regulations; Food control management; Inspection services; Food monitoring and epidemiological Data; and Communication, information, education, and training as recommended by the FAO [33,34].

There is great diversity in these countries for the establishment and effectiveness of food safety legislation. For example, the Saudi Food and Drug Authority (SFDA) was established in 2003 as an independent body directly reporting to the Prime Minister with the responsibility to regulate, oversee, and control food, drug, medical devices, and the Gulf Standardization Organization (GSO) was established within the Gulf countries with the aim to harmonize the Standards and Technical Regulations of member countries based on Codex Alimentarius and in efforts to meet the requirements the Technical Barriers to Trade (TBT) and the Sanitary and Phytosanitary (SPS) Measures Agreement under the World Trade Organization (WTO). In contrast, Lebanon is still working on the legislation required to enter the WTO while facing many challenges of sectarian and political turmoil, the failure of economic growth, and massive influx of refugees from Syria. Lebanon passed its food safety law as late as 2016 after drafts had been discussed as early as 2004, with a view to establish public governance of food safety. Before the publication of the 2016 Food Safety Law, there were nine government agencies dealing with food safety, but there was no coordination among them [35]. The Food Safety Lebanese Commission (FSLC) was given responsibilities under the Law to build-up the system of food safety and sub-systems in all the ministries and organizations, and to establish by-laws and policies that would be implemented under the Council of Ministers. The FSLC was also tasked with developing education and training of professionals through academic institutions for the food industry, setting up the means for well-trained inspectors to monitor the food supply and accreditations for new laboratories. The challenge for the FSLC will be for its recommendations to be accepted by existing food safety agencies and at the cabinet level, especially today under conditions of civil unrest and economic hardship. These issues are not unique to Lebanon where weak governments combined with powerful external lobbyists can delay or minimize effective prevention and control measures for food safety. Another example is in Palestine, where the current food safety legislations are not harmonized with international standards [30]. 

Priority for food safety sometimes only occurs after a number of food scares are sufficient to mobilize the public to demand change. It was not until January 2017 when the Egyptian Parliament established the National Food Safety Authority (NFSA) to exclusively assume the responsibilities and jurisdiction of all ministries, public institutions, government agencies, and municipalities in relation to supervision over the handling of foodstuff with the aim to improve the regulatory oversight and efficiency in the food system. This is one step beyond the Lebanese FSLC, which has to collaborate with other agencies. Nevertheless, an agency having been given complete authority does not necessarily translate into safer food for the residents of Egypt or for products exported to other countries. Although the United Arab Emirates (UAE) has a federal law on food safety passed in 2016, food safety control in the emirates of Dubai and Sharjah is managed at the municipal level, and Dubai has established an international reputation for hosting the annual Dubai International Food Safety Conferences well before the law was promulgated. For the most part, food-borne diseases in the Eastern Mediterranean Region are still generally not well understood because of the ineffective food-borne illness surveillance and many illness cases are perceived as mild and self-limiting or unverified due to gaps in detection, surveillance and reporting by authorities. This partly reflects on the commitment of agencies to support sufficient numbers of qualified inspectors and testing laboratories to monitor the food supply. Where surveillance exists, reportable diseases in many of these countries tend to include food poisoning as a catch-all rather than list specific food-borne diseases, and the agent is not necessarily required to be identified during an investigation. 

PulseNet Middle East was established in 2006 with 10 countries in the Eastern Mediterranean Region participating for molecular surveillance of food-borne infectious diseases using pulsed-field gel electrophoresis (PFGE), but it has yet to play a large role in identifying agents and factors contributing to illness, and recalling contaminated products in the Region. Since Whole Genomic Sequencing (WGS) has largely taken over from PFGE testing in Western nations, it remains to be seen if MENA countries can utilize molecular surveillance more effectively for improving food safety for the public. Even if a country has the capability to use WGS, it may not be effective unless linked to an overall surveillance and management structure such as a National Food Control System [30]. Aggressive closure of food facilities by inspectors, sometimes in collaboration with the local police force, can occur after publicly-reported food poisonings, or violations identified during an inspection, such as ‘eating spoiled foods’ to be used as deterrents for perceived compliance failures. During these closures, owners are forced financially to let go their employees temporarily. Unfortunately, these limited investigations often fail to determine the source of causative agents or to recommend educational advice to avoid future to risk behavior. Fines can also be imposed on the owners of these facilities which may be encouraged as an important source of revenue for cash-strapped public health agencies. It is difficult to ascertain the burden of food-borne diseases in many Middle Eastern countries especially when rural areas may see less inspection than in urban centers, and these are more likely to be underestimated than in western nations. As the authors state, inspection activities in the majority of the countries follow a reactive approach relying on end-products sampling, focusing on sanitation, personal hygiene, food labels instead of risk-based preventive approaches.

Governments in MENA countries tend not to be directly involved in promoting food safety training, and where these exist, they are the responsibility of the private sector, or are sponsored by non-governmental organizations, for programs like understanding and developing targeted hazard analysis critical control point (HACCP) plans. A driver for training for safe food is more linked to satisfying importers in other countries. The 2011 U.S. Food Safety Modernization Act [36] gives the Food and Drug Administration the authority to require exporters to the United States to satisfy certain criteria before they are allowed to export products, such as requiring that high-risk imported foods be accompanied by a credible third party certification or other assurance of compliance as a condition of entry into the U.S. High-risk products include those implicated in food-borne illnesses such as sesame-seed based tahini and other seed products, nuts, berries as well as meat and dairy foods. The authors conclude that there has to be more research and scientific outputs to understand the local food chain systems, to strengthen the food-borne disease surveillance systems, and to further develop capacity building programs to build NFCS using a risk-based approach to prevention and control of food-borne disease.

Several countries in the MENA region have made substantial efforts in improving their food safety systems and in some cases, in unifying the food control activities under one central agency. However, many challenges are still encountered due to ineffective surveillance systems, lack of communication among stakeholders, and limited, sometimes absent, food control functions along the food supply chain. MENA countries have limited capacity to enforce the law and implement food safety policies on a large scale and foster inter-communications among stakeholders.

These guidelines provide information for government agencies to assist in the development of national food control systems (NFCS) and to promote effective collaboration between stakeholders involved in the management and control of food safety and quality. However, different risk management decisions could be made at national levels according to different criteria and different ranges of risk management options.

In conclusion, these five papers add to our knowledge of how to understand why preventing and controlling food-borne illness is so difficult. Consumers and the public in general react to broadcast news and nowadays social media, as well as their base culture, for setting their anchoring of how they perceive risks of illness from eating specific food items. Food scares and epidemics/pandemics often reset perceptions which may last for many years depending on how much coverage the public is exposed to. The science through risk assessments and epidemiological investigations can weigh the risks of food-borne illness to a population to help governments and industry react appropriately with interventions and advice, but how much is absorbed and acted on by the public depends on the following factors: (1) trust in the responsible agency or company; (2) the acceptable level of risk communication for lay audiences; (3) the variety of communication approaches, and the duration of the messages over an extended period of time.
